# Prognostic Implications of Type 2 Diabetes Mellitus in Heart Failure with Mildly Reduced Ejection Fraction

**DOI:** 10.3390/jcm13030742

**Published:** 2024-01-27

**Authors:** Tobias Schupp, Mohammad Abumayyaleh, Kathrin Weidner, Felix Lau, Marielen Reinhardt, Noah Abel, Alexander Schmitt, Jan Forner, Niklas Ayasse, Thomas Bertsch, Muharrem Akin, Ibrahim Akin, Michael Behnes

**Affiliations:** 1 Department of Cardiology, Angiology, Haemostaseology and Medical Intensive Care, University Medical Centre Mannheim, Medical Faculty Mannheim, Heidelberg University, 68167 Mannheim, Germany; 2 Vth Department of Medicine (Nephrology, Hypertensiology, Endocrinology, Rheumatology, Pneumology), Transplant Center Mannheim, Medical Faculty Mannheim, University Hospital Mannheim, Heidelberg University, 68167 Mannheim, Germany; 3 Institute of Clinical Chemistry, Laboratory Medicine and Transfusion Medicine, Nuremberg General Hospital, Paracelsus Medical University, 90419 Nuremberg, Germany; 4 Department of Cardiology, St. Josef-Hospital, Ruhr-Universität Bochum, 44791 Bochum, Germany

**Keywords:** heart failure with mildly reduced ejection fraction, HFmrEF, diabetes mellitus, mortality

## Abstract

Background: Data regarding the characterization and outcomes of diabetics with heart failure with a mildly reduced ejection fraction (HFmrEF) is scarce. This study investigates the prevalence and prognostic impact of type 2 diabetes in patients with HFmrEF. Methods: Consecutive patients with HFmrEF (i.e., left ventricular ejection fraction 41–49% and signs and/or symptoms of HF) were retrospectively included at one institution from 2016 to 2022. Patients with type 2 diabetes (dia-betics) were compared to patients without (i.e., non-diabetics). The primary endpoint was all-cause mortality at 30 months. Statistical analyses included Kaplan–Meier, multivariable Cox regression analyses and propensity score matching. Results: A total of 2169 patients with HFmrEF were included. The overall prevalence of type 2 diabetes was 36%. Diabetics had an increased risk of 30-months all-cause mortality (35.8% vs. 28.6%; HR = 1.273; 95% CI 1.092–1.483; *p* = 0.002), which was confirmed after multivariable adjustment (HR = 1.234; 95% CI 1.030–1.479; *p* = 0.022) and propensity score matching (HR = 1.265; 95% CI 1.018–1.572; *p* = 0.034). Diabetics had a higher risk of HF-related rehospitalization (17.8% vs. 10.7%; HR = 1.714; 95% CI 1.355–2.169; *p* = 0.001). Finally, the risk of all-cause mortality was increased in diabetics treated with insulin (40.7% vs. 33.1%; log-rank *p* = 0.029), whereas other anti-diabetic pharmacotherapies had no prognostic impact in HFmrEF. Conclusions: Type 2 diabetes is common and independently associated with adverse long-term prognosis in patients with HFmrEF.

## 1. Introduction

The risk of heart failure (HF) has been shown to be twice as high in men and five times higher in women suffering from diabetes mellitus compared to non-diabetics [1]. Ongoing demographic changes have led to an increasing prevalence of obesity even in HF, accompanied by an increasing number of patients with HF and type 2 diabetes mellitus (DM II) is consistently increasing [2,3,4]. DM II sustaines the development of coronary artery disease (CAD), as well as insulin resistance, glucose toxicity, vascular and microcirculatory dysfunction, inflammation and the activation of the renin–angiotensin–aldosterone system (RAAS) [5,6,7]. Of note, the prevalence of HF is also increased in the absence of significant CAD. This so-called “diabetic cardiomyopathy” may specifically lead to HF with preserved ejection fraction (HFpEF), whereas left ventricular (LV) dysfunction is typically present in advanced stages of DM II [8,9].

Recently, following the revision of the European guidelines for the management of HF, a third category—patients with HF with mildly reduced ejection fraction (HFmrEF)—was introduced in addition to patients with HF with reduced (HFrEF) and preserved ejection fraction (HFpEF) [10]. This subgroup of HF patients is largely unexplored in clinical studies, leading to limited guideline recommendations for these patients. Recently, a comparable risk of all-cause mortality in patients with DM was demonstrated in a study including 2258 patients with acute HF stratified by left ventricular ejection fraction (LVEF) (i.e., HFrEF, HFmrEF and HFpEF) [11]. Furthermore, the prognosis of patients with and without DM did not differ in patients with acute HF, irrespective of the HF category, while 962 patients suffered from HFmrEF [12]. In contrast, DM was shown to impair the long-term prognosis of patients with acute coronary syndrome (ACS) and HFmrEF [13], whereas no association with all-cause mortality was observed in patients with cardiogenic shock [14]. 

However, studies investigating the prognostic value of concomitant DM II in patients with HFmrEF are inconclusive and predominantly restricted to specific subgroups (i.e., acute HF, ACS and acute myocardial infarction (AMI)) [15], whereas the prognostic impact of DM II in HFmrEF is still unclear. Accordingly, data investigating predictors of adverse prognosis in patients with HFmrEF stratified by the presence or absence of diabetes and the prognostic role of anti-diabetic therapies is rare. Therefore, the present study sought to investigate (1) the prognostic impact of DM II in patients with HFmrEF, (2) predictors of all-cause mortality and HF-related rehospitalization and (3) anti-diabetic pharmacotherapies in HFmrEF.

## 2. Materials and Methods

### 2.1. Study Patients, Design and Data Collection 

For the present study, all consecutive patients hospitalized from January 2016 to December 2022 with HFmrEF at one university medical center were included, as recently published [16]. Using the electronic hospital information system, all relevant clinical data related to the index event were documented, such as baseline characteristics, vital signs on admission, prior medical history, prior medical treatment, length of index hospital and intensive care unit (ICU) stay, laboratory values, data derived from all non-invasive or invasive cardiac diagnostics and device therapies (such as echocardiographic data, coronary angiography and data derived from prior or newly implanted cardiac devices). Every revisit at the outpatient clinic or rehospitalization related to HF or adverse cardiac events was documented until the end of the year 2022. Heart rate (HR) was measured using 12-lead electrocardiography (ECG).

The present study is derived from the “Heart Failure with Mildly Reduced Ejection Fraction Registry” (HARMER), representing a retrospective single-center registry including all consecutive patients with HFmrEF hospitalized at the University Medical Center Mannheim (UMM), Germany (clinicaltrials.gov identifier: NCT05603390). The registry was carried out according to the principles of the Declaration of Helsinki and was approved by the Medical Ethics Committee II of the Medical Faculty Mannheim, University of Heidelberg, Germany (ethical approval code: 2022-818).

### 2.2. Inclusion and Exclusion Criteria

All consecutive patients ≥ 18 years of age hospitalized with HFmrEF at one institution were included. Patients < 18 years of age were excluded. The diagnosis of HFmrEF was determined according to the “2021 ESC Guidelines for the diagnosis and treatment of acute and chronic heart failure” [10]. Accordingly, all patients with LVEF 41–49% and symptoms and/or signs of HF were included. The presence of elevated aminoterminal prohormone levels in brain natriuretic peptide (NT-proBNP) and other evidence of structural heart disease were considered to make the diagnosis more likely but were not mandatory for diagnosis of HFmrEF. Transthoracic echocardiography was performed by cardiologists during routine clinical care; they were blinded to the final study analysis in accordance with current European guidelines [17]. For the present study, all echocardiographic examinations and reports were reassessed post hoc by two independent cardiologists, whereas important echocardiographic measurements were reassessed. The presence of right ventricular dysfunction was defined as a tricuspid annular plane systolic excursion (TAPSE) < 18 mm.

### 2.3. Risk Stratification

For the present study, risk stratification was performed according to the presence or absence of DM II. Documentation of new-onset or pre-existent DM II was derived from documented medical history within the electronic hospital information system. DM II was defined in the presence of glycated hemoglobin A1c (HbA1c) ≥ 6.5%, fasting plasma glucose levels ≥ 126 mg/dL or 2 h post-load plasma glucose levels ≥ 200 mg/dL in accordance with established guidelines [18]. Patients with DM other than type II were excluded from the present study.

### 2.4. Study Endpoints

The primary endpoint was long-term all-cause mortality. Long-term was defined as the median time of clinical follow-up in months (i.e., 30 months). Secondary endpoints comprised in-hospital all-cause mortality, all-cause mortality at 12 months, rehospitalization for worsening HF at 30 months and cardiac rehospitalization, acute myocardial infarction (AMI), stroke, coronary revascularization and major adverse cardiac and cerebrovascular events (MACCE) at long-term follow-up, as well as changes in LVEF and NT-pro BNP levels during the follow-up period. All-cause mortality was documented using the electronic hospital information system and by directly contacting state resident registration offices (‘bureau of mortality statistics’). HF-related hospitalization was defined as a rehospitalization due to worsening HF requiring intravenous diuretic therapy. HF-related rehospitalization comprised patients with hospitalization due to worsening HF as the primary cause, as a result of another cause but associated with worsening HF at the time of admission or as a result of another cause but complicated by worsening HF during its cause. Cardiac rehospitalization was defined as rehospitalization due to a primary cardiac condition, including worsening HF, AMI, coronary revascularization and symptomatic atrial or ventricular arrhythmias. MACCE were defined as a composite of all-cause mortality, coronary revascularization, non-fatal AMI and non-fatal stroke. Time-trend sub-analyses evaluated the course of LVEF, NT-proBNP levels and estimated glomerular filtration rate (eGFR) at follow-up every 6 months in patients comparing diabetics and non-diabetics. Here, all available echocardiographic examinations being investigated during routine care either within (re)hospitalization or ambulatorily in the outpatient clinic at our institution were documented at six-month intervals (0–6, 6–12, 12–18, 18–24 and 24–30 months). 

### 2.5. Statistical Methods

Quantitative data is presented as mean ± standard error of mean (SEM), median and interquartile range (IQR) and ranges depending on the distribution of the data. They were compared using the Student’s *t*-test for normally distributed data or the Mann–Whitney U test for non-parametric data. Deviations from a Gaussian distribution were tested using the Kolmogorov–Smirnov test. Qualitative data is presented as absolute and relative frequencies and were compared using the Chi-square test or the Fisher’s exact test, as appropriate. Kaplan–Meier analyses were performed comparing diabetics and non-diabetics, as well as stratifying by the need for insulin treatment in diabetics (IDDM vs. NIDDM). Kaplan–Meier analyses were performed regarding the risk of all-cause mortality and HF-related rehospitalization. With regard to the risk of rehospitalization, only patients surviving index hospitalizations were included. Univariable hazard ratios (HRs) were given together with 95% confidence intervals. The prognostic impact of DM II was thereafter investigated within multivariable Cox regression models using the “forward selection” option. LVEF, NT-pro BNP levels and eGFR were compared among patients with and without DM II at 6-month intervals following index hospitalization using the Student’s *t*-test or the Mann–Whitney U test. Related to the all-comers study design, additional propensity score matching was applied to account for the heterogenous distribution of baseline characteristics and comorbidities comparing diabetics and non-diabetics. Propensity score matching analyses were applied for the comparison of diabetics compared to non-diabetics, including the entire study cohort and applying a non-parsimonious multivariable logistic regression model. Propensity scores were created according to the presence of the following independent variables: age, sex, body mass index (BMI), prior CAD, prior AMI, prior congestive HF, prior decompensated HF < 12 months, chronic kidney disease, peripheral artery disease, malignancies, chronic obstructive pulmonary disease (COPD), arterial hypertension, hyperlipidemia, smoking status, AMI on admission, HF etiology, acute decompensated heart failure (ADHF), NYHA functional class, LVEF, TAPSE, the presence or absence of aortic stenosis, regurgitation and mitral or tricuspid regurgitation, eGFR and hemoglobin on admission. Based on the propensity score values counted using logistic regression, for each patient, one patient in the control group with a similar propensity score value was found (accepted difference of propensity score value < 1%). Within the propensity score-matched subgroup, the Kaplan–Meier method was applied, and univariable HRs were given together with 95% confidence intervals. Thereafter, multivariable Cox regression analyses were performed stratified by the presence or absence of DM II to investigate predictors of prognosis in diabetics and non-diabetics. Finally, additional multivariable Cox regression analyses were performed, focusing on anti-diabetic therapies.

The results of all statistical tests were considered significant at *p* ≤ 0.05. SPSS (Version 28, IBM, Armonk, NY, USA) was used for statistics.

## 3. Results

### 3.1. Study Population 

From 2016 to 2022, 2228 patients with HFmrEF were hospitalized at our institution. A total of 44 patients with incomplete follow-up and 15 patients with type 1 diabetes were excluded. The final study cohort comprised 2169 patients with HFmrEF with an overall prevalence of DM II of 36% (*n* = 784). Diabetics were older (median age 77 vs. 74 years; *p* = 0.001), had higher rates of prior CAD (49.7% vs. 36.0%; *p* = 0.001), chronic kidney disease (40.2% vs. 25.7%; *p* = 0.001) and congestive HF (39.7% vs. 30.7%; *p* = 0.001), with a higher proportion of patients being hospitalized for acute decompensated HF < 12 months (13.5% vs. 9.3%; *p* = 0.002) (Table 1; left panel). Furthermore, other cardiovascular risk factors included arterial hypertension (90.7% vs. 70.5%; *p* = 0.001) and hyperlipidemia (38.3% vs. 25.7%; *p* = 0.001). Furthermore, diabetics had higher rates of acute decompensated HF at index hospitalization (28.8% vs. 18.3%; *p* = 0.001). In contrast, the rates of atrial fibrillation (43.8% vs. 41.2%; *p* = 0.253) and cardiopulmonary resuscitation (2.6% vs. 2.4%; *p* = 0.807) did not significantly differ in both groups.

Ischemic cardiomyopathy was the most common HF etiology in both groups, with a higher prevalence in diabetics (64.9% vs. 53.5%; *p* = 0.001) (Table 2; left panel). Diabetics had more advanced stages of NYHA functional class (i.e., NYHA III and IV: 35.5% vs. 23.3%; *p* = 0.001). With regard to echocardiographic parameters, especially TAPSE (median 20 vs. 20 mm; *p* = 0.152) and valvular heart diseases did not differ in patients with or without diabetes. In contrast, a higher proportion of diabetics had three-vessel CAD (55.7% vs. 32.5%; *p* = 0.001), along with a higher rate of coronary artery bypass grafting (CABG) (12.4% vs. 5.4%; *p* = 0.001) and coronary chronic total occlusion (CTO) (18.3% vs. 9.5%; *p* = 0.001). Subsequently, a higher proportion of diabetics was sent to CABG following index coronary angiography (9.6% vs. 3.5%; *p* = 0.001). With regard to laboratory data, diabetics had higher creatinine levels (1.20 mg/dL vs. 1.02 mg/dL; *p* = 0.001) and lower hemoglobin levels (median 12.0 g/dL vs. 12.6 g/dL; *p* = 0.001). Finally, a higher proportion of diabetics was treated with angiotensin receptor blockers (ARB) (28.7% vs. 20.6%; *p* = 0.001) and aldosterone antagonists (16.0% vs. 12.8%; *p* = 0.046). With regard to diabetes-related treatment, most diabetics were treated with insulin (38.0%), followed by metformin (36.7%) (Table 2; left panel).

### 3.2. Prognostic Value of Type 2 Diabetes in Patients with HFmrEF

**Figure 1:**
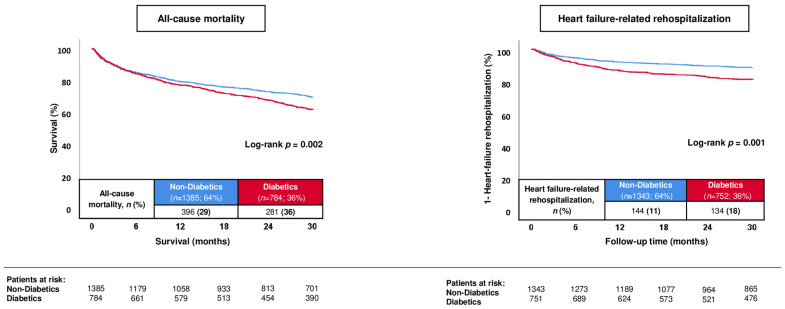
Prognostic impact of type 2 diabetes mellitus in patients with HFmrEF with regard to 30-month all-cause mortality (**left panel**) and HF-related rehospitalization (**right panel**) within the entire study cohort.

During a median follow-up of 30 months, the primary endpoint of all-cause mortality occurred in 35.8% of diabetics and in 28.6% of non-diabetics (log-rank *p* = 0.002) (Figure 1; left panel). Diabetics were associated with a higher risk of 30-month all-cause mortality compared to non-diabetics (HR = 1.273; 95% CI 1.092–1.483; *p* = 0.002). Furthermore, the risk of rehospitalization for worsening HF was higher in diabetics (17.8% vs. 10.7%; log-rank *p* = 0.001; HR = 1.714; 95% CI 1.355–2.169; *p* = 0.001) (Figure 1; right panel). In line with this, the risks of cardiac rehospitalization (25.7% vs. 19.8%; HR = 1.344; 95% CI 1.108–1.606; *p* = 0.002), revascularization (8.4% vs. 5.7%; HR = 1.467; 95% CI 1.058–2.059; *p* = 0.022), AMI (4.7% vs. 2.2%; HR = 2.169; 95% CI 1.326–3.548; *p* = 0.002) and MACCE (44.1% vs. 35.2%; HR = 1.298; 95% CI 1.131–1.489; *p* = 0.001) were higher in diabetics than in non-diabetics (Table 3; endpoints).

### 3.3. Propensity Score Matching

**Figure 2:**
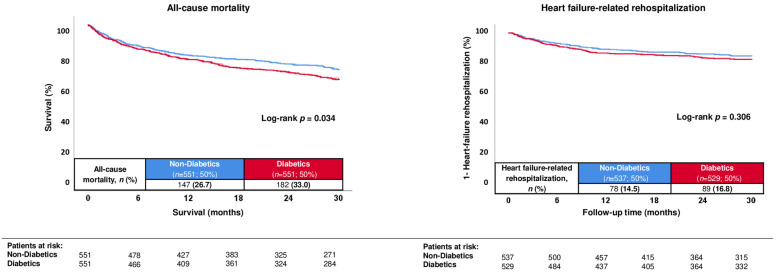
Prognostic impact of type 2 diabetes mellitus in patients with HFmrEF with regard to 30-month all-cause mortality (**left panel**) and HF-related rehospitalization (**right panel**) after propensity score matching.

Even after propensity score matching (*n* = 551 diabetics and non-diabetics), especially age, sex and vital signs on admission were equally distributed in both groups, along with similar rates of prior congestive HF and decompensated HF < 12 months (Table 1; right panel). In line, the distribution of HF etiologies and NYHA functional class did not differ in both groups (Table 2; right panel). Even after propensity score matching, the risk of all-cause mortality at 30 months was still higher in diabetics than in non-diabetics (33.0% vs. 26.7%; log-rank *p* = 0.034; HR = 1.265; 95% CI 1.018–1.572; *p* = 0.034) (Figure 2; left panel). In contrast, the risk of HF-related rehospitalization at 30 months did not differ in diabetics and non-diabetics after propensity score matching (16.8% vs. 14.5%; log-rank *p* = 0.306; HR = 1.172; 95% CI 0.865–1.589; *p* = 0.306) (Figure 2; right panel).

### 3.4. Changes in LVEF, NT-proBNP Levels and eGFR in Diabetics and Non-Diabetics

**Figure 3:**
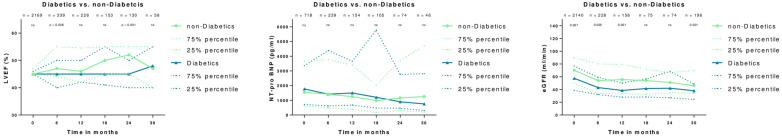
Changes in LVEF (**left panel**), NT-pro BNP levels (**middle panel**) and eGFR (**right panel**) among diabetics and non-diabetics during 30-month follow-up. Data are presented as median and interquartile range (IQR).

During a follow-up period of 30 months, LVEF was lower in diabetics than in non-diabetics at 6 months (median 45% vs. 47%; *p* = 0.006) and 24 months (median 45% vs. 52%; *p* = 0.001), whereas LVEF values did not differ in both groups at 12, 18 and 20 months of follow-up (Figure 3; left panel). In contrast, NT-proBNP levels were comparable in diabetics and non-diabetics at all time points during follow-up (Figure 3; middle panel). Finally, eGFR values were lower in diabetics during index hospitalization (median 57.6 mL/min vs. 70.2 mL/min; *p* = 0.001), as well as at 6 months (median 43.0 mL/min vs. 54.0 mL/min; *p* = 0.005), 12 months (median 38.5 mL/min vs. 55.5 mL/min; *p* = 0.001) and 30 months thereafter (median 38.0 mL/min vs. 46.0 mL/min; *p* = 0.001) (Figure 3; right panel).

### 3.5. Predictors of Prognosis in Diabetics and Non-Diabetics

In diabetics, the risk of all-cause mortality at 30 months was higher in patients with higher age, higher creatinine levels and in patients with acute decompensated HF, whereas higher hemoglobin levels were associated with improved survival rates (Table 4). In contrast, an increased risk of all-cause mortality in non-diabetics was observed in patients with higher age, males and in patients with acute decompensated HF, whereas creatinine levels were not associated with outcomes. Furthermore, a higher body mass index, the presence of AMI and hyperlipidemia were associated with improved survival in patients without diabetes.

### 3.6. Prognostic Impact of Diabetes-Related Treatment

**Figure 4:**
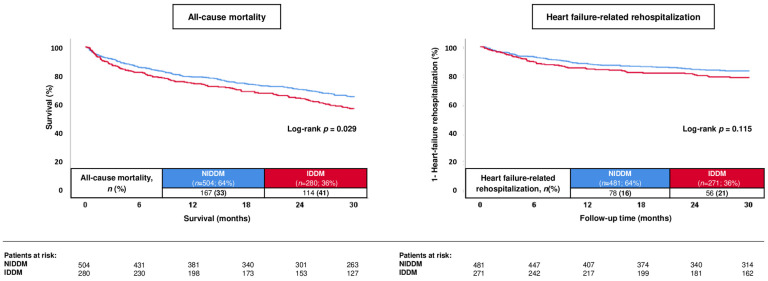
Prognostic impact of insulin-dependent diabetes in type 2 diabetics with HFmrEF with regard to 30-month all-cause mortality (**left panel**) and HF-related rehospitalization (**right panel**).

In patients with type 2 diabetes, 38.0% of patients had IDDM. Patients with IDDM had a higher risk of 30-months all-cause mortality compared to patients with NIDDM (40.7% vs. 33.1%; log-rank *p* = 0.029) (Figure 4; left panel). In contrast, the risk of HF-related rehospitalization did not differ in patients with IDDM and NIDDM (log-rank *p* = 0.115). After multivariable adjustment, patients with IDDM were still associated with a higher risk of 30-months all-cause mortality compared to NIDDM patients (HR = 1.332; 95% CI 1.018–1.742; *p* = 0.047), whereas other anti-diabetic pharmacotherapies had no prognostic impact on the risk of all-cause death in diabetics (Table 5). 

## 4. Discussion

The present study differentiates the prevalence and long-term prognostic impact of type 2 diabetes in patients with HFmrEF using a large, retrospective single-center registry from 2016 to 2022. This data suggests that DM II represents one of the most common non-cardiac comorbidities, with an overall prevalence of 36% in patients with HFmrEF. Diabetics had an increased risk of 30-months all-cause mortality and HF-related rehospitalization. The increased risk of all-cause mortality was still evident after multivariable adjustment and propensity score matching. In line with this, the rates of coronary revascularization, AMI and MACCE were higher in diabetics. Finally, patients with IDDM had an increased risk of 30-months all-cause death compared to those with NIDDM, whereas other anti-diabetic pharmacotherapies had no prognostic impact on the prognosis of diabetics with HFmrEF.

DM II represents one of the most common cardiovascular risk factors, affecting about 40% of patients hospitalized for HF [19,20]. Diabetics are associated with an increased risk of all-cause mortality in various clinical conditions, including atrial fibrillation and ventricular tachyarrhythmias [21,22,23]. However, whether DM itself represents an independent predictor of mortality in patients with HF remains controversial. Data from the OPTIMIZE-HF trial suggested that the presence of DM was not independently associated with the risk of in-hospital and mortality at follow-up in more than 48,000 patients with HF and a mean LVEF of 39%. Surprisingly, follow-up mortality was even lower in patients with DM in their study within the subgroup of patients without LV dysfunction, which may be attributed to improved guideline-recommended therapies in patients with concomitant DM [19]. In contrast, data from the EVEREST trial suggested an increased risk of all-cause mortality and hospitalization for HF in diabetics, including 4133 patients with HF and LVEF < 40% [20]. Adverse prognosis diabetics is supported by the Korean Acute Heart Failure registry, including 5625 patients with acute HF; however, this association was only evident for patients with HFrEF, whereas no prognostic value of diabetes was demonstrated in HFmrEF and HFpEF. However, only 877 patients in their study suffered from HFmrEF [24]. To the best knowledge of the authors, the present study is the largest to investigate the prognostic impact of DM in consecutive patients with HFmrEF, suggesting an increased risk of adverse long-term prognosis, including all-cause mortality, hospitalization for worsening HF, coronary revascularization, AMI and MACCE at 30 months. The adverse prognostic impact of DM was still evident after multivariable adjustment and propensity score matching, suggesting an independent association of DM with adverse outcomes in patients with HFmrEF. 

Of note, the characteristics of patients with and without DM were shown to differ significantly in HF studies, including a higher rate of ischemic etiology and chronic kidney disease in individuals with concomitant DM [19,20]. Although ischemic cardiomyopathy was shown to be the most common etiology leading to HFmrEF, the distribution of comorbidities differed among patients with HFmrEF, HFrEF and HFpEF [25,26,27]. Thus, DM was especially shown to be the most common comorbidity with a prevalence of 43% in patients with HFmrEF [25,28]. Interestingly, Dries et al. suggested that the prognostic role of concomitant DM may vary among different HF etiologies. Within a post hoc analysis of the SOLVD trial, it was suggested that DM may specifically impair prognosis in patients with ischemic etiology, whereas DM had no prognostic impact in patients with non-ischemic cardiomyopathy [29]. In the present study, almost two-thirds of patients suffered from ischemic cardiomyopathy, with higher rates of three-vessel CAD, CABG and coronary CTO. Alongside, a higher proportion of diabetics was sent to CABG following index coronary angiography, with subsequent higher rates of AMI and coronary revascularization during follow-up.

Furthermore, diabetes-related microangiopathy was shown to contribute to increased mortality rates in diabetics. From this perspective, diabetic neuropathy was shown to increase the risk of stroke even more compared to diabetic nephropathy [30]. A meta-analysis including 25 studies and 2935 patients demonstrated decreased heart rate variability (HVR) in patients with concomitant DM [31]. Even resting HR was demonstrated to be an independent predictor of diabetes-related mortality in 1877 elderly diabetics [32]. However, the present study did not find an association between HR on admission in patients with HFmrEF, irrespective of the presence of concomitant DM. In line with this, Hansen et al. did not find an association between changes in HR and HRV in 4166 people with and without dysglycemia [33]. Furthermore, Mayyas et al. suggested that cardiovascular disease and chronic kidney disease specifically represent the strongest predictors of diabetes-related mortality [34]. In the present study, the presence of impaired renal function was associated with an increased risk of all-cause mortality in patients with concomitant DM. eGFR values were lower in diabetics at 6, 12 and 30 months following index hospitalization, which may be in line with the study by Mayyas et al., who suggested that the presence of chronic kidney disease may predict adverse outcomes. The Studies of Left Ventricular Dysfunction (SOLVD) trial suggested DM was an independent predictor of worsening renal function in 6758 patients with congestive HF, whereas worsening renal function specifically predicted adverse outcomes [35].

Besides the risk of microvascular diseases, DM is associated with important comorbidities, especially arterial hypertension, hyperlipidemia and the presence and extent of CAD [36]. Especially the risk of obstructive sleep apnea syndrome (OSAS) and metabolic syndrome is increased [37]. The presence of OSAS was shown to be associated with adverse outcomes, including the risk of developing chronic kidney disease and tricuspid or aortic regurgitation in patients with HFmrEF [38]. In the present study, the distribution of BMI as well as obesity-related comorbidities were similar after propensity score matching, suggesting an independent association between diabetes and the risk of all-cause mortality in patients hospitalized with HFmrEF.

Data focusing on the prognostic impact of diabetes-related pharmacotherapies in patients with concomitant HFmrEF patients is scarce. In the EVEREST trial, no prognostic difference concerning diabetes-related therapeutic strategies (i.e., diet alone, oral treatment or insulin) with regard to the risk of all-cause mortality or HF-related hospitalization was observed [20]. In contrast, an increased risk of all-cause death was observed among patients with IDDM, including 496 patients with DM with HFrEF following AMI [15]. Impaired outcomes in patients receiving insulin were specifically observed in patients with lower HbA1c within the Korean Acute Heart Failure registry [39]. The present study specifically suggested that patients with IDDM had an increased risk of all-cause death compared to patients with NIDDM, which was confirmed after multivariable adjustment. This may be in line with the adverse prognosis in patients with insufficient glycemic control in patients with orally treated DM [24]. 

However, in the present study, other diabetes-related pharmacotherapies had no prognostic impact on the risk of all-cause mortality. Besides the treatment with insulin, treatment with sodium glucose transporter 2 (SGLT2) inhibitors has gained more importance related to randomized controlled trials for HF patients with and without concomitant DM II [40,41,42,43]. As a result, treatment with SGLT2 inhibitors in patients with DM II increased to 12% in 2018 in the Swedish HF registry [44] and was upgraded to a class 1A indication for patients with HFmrEF in 2023 [45]. However, in the present study, only 8.9% of diabetics with HFmrEF were treated with SGLT2 inhibitors from 2016 to 2022, which may explain the lack of a mortality benefit in our study. Further studies are warranted to investigate the prognostic impact of anti-diabetic therapies in patients with HFmrEF. Furthermore, a very low proportion of patients was treated with angiotensin receptor-neprilysin inhibitors (ARNI) in the present study, which may further reduce the risk of HF-related rehospitalization [46]. However, this may be related to the limited evidence of ARNI in patients with HFmrEF [10,45,47]. Even though the proportion of patients with optimal medical HF treatment will increase in patients with HFmrEF due to the upgrade of SGLT2 inhibitors in the revised European HF guidelines in 2023 [45], further studies are needed concerning the use of ARNI in patients with HFmrEF.

## 5. Study Limitations

This study has several limitations. Due to the retrospective and single-center study design, the results may be influenced by measured and unmeasured confounding, although we tried to adjust for potential confounding using multivariable risk prediction models and propensity score matching. HF-related and cardiac rehospitalizations were assessed at our institution only. For the present study, no information on HbA1c values during follow-up or HbA1c variability was available [48]. For the present study, the rates of holter ECG were low. Therefore, no further sub-analyses regarding the prognostic impact of diabetic neuropathy assessed by HR changes and HRV were performed. The prognostic impact of worsening renal function was beyond the scope of this manuscript. Finally, the causes of death beyond index hospitalization at long-term follow-up were not available for the present study.

## 6. Conclusions

Type 2 diabetes represents a common cardiovascular risk factor in patients with HFmrEF, with an overall prevalence of 36%. The presence of type 2 diabetes was independently associated with an increased risk of 30-month all-cause mortality. Finally, patients with IDDM had an increased risk of all-cause mortality compared to patients with NIDDM, whereas other anti-diabetic pharmacotherapies had no prognostic impact in patients with HFmrEF.

## Figures and Tables

**Table 1:** Baseline characteristics.

	Without Propensity Score Matching					With Propensity Score Matching				
	Non-Diabetics (*n* = 1385)		Diabetics (*n* = 784)		*p*-Value	Non-Diabetics (*n* = 551)		Diabetics (*n* = 551)		*p*-Value
**Age**, median (IQR)	74	(62–82)	77	(68–83)	**0.001**	76	(66–83)	76	(67–83)	0.535
**Male sex**, *n* (%)	868	(62.7)	534	(64.1)	**0.011**	383	(69.5)	373	(67.7)	0.516
**Body mass index**, kg/m^2^, median (IQR)	25.6	(23.2–29.3)	29.0	(25.2–32.6)	**0.001**	27.1	(24.3–31.2)	28.0	(24.8–31.1)	0.214
**SBP**, mmHg, median (IQR)	140	(124–160)	144	(127–167)	**0.001**	145	(125–164)	144	(125–166)	0.883
**DBP**, mmHg, median (IQR)	80	(70–90)	78	(67–90)	**0.029**	80	(70–90)	78	(67–90)	**0.049**
**Heart rate**, bpm, median (IQR)	81	(68–96)	80	(68–93)	0.110	80	(67–92)	80	(69–93)	0.677
**Medical history**, *n* (%)										
Coronary artery disease	499	(36.0)	390	(49.7)	**0.001**	268	(48.6)	256	(46.5)	0.469
Prior myocardial infarction	291	(21.0)	225	(28.7)	**0.001**	159	(28.9)	147	(26.7)	0.420
Congestive heart failure	425	(30.7)	311	(39.7)	**0.001**	200	(36.3)	196	(35.6)	0.802
Decompensated heart failure < 12 months	129	(9.3)	106	(13.5)	**0.002**	67	(12.2)	58	(10.5)	0.393
Prior ICD	25	(1.8)	17	(2.2)	0.555	13	(2.4)	11	(2.0)	0.680
Primary prevention	19	(76.0)	9	(52.9)	0.120	10	(76.9)	8	(72.7)	0.334
Secondary prevention	6	(24.0)	8	(47.1)		3	(23.1)	3	(67.3)	
Prior s-ICD	8	(0.6)	1	(0.1)	0.117	2	(0.4)	0	(0.0)	0.157
Primary prevention	5	(62.5)	0	(0)	0.236	2	(100)	0	(0.0)	0.800
Secondary prevention	3	(37.5)	1	(100.0)		0	(0.0)	0	(0.0)	
Chronic kidney disease	356	(25.7)	315	(40.2)	**0.001**	198	(35.9)	182	(33.0)	0.311
Peripheral artery disease	112	(8.1)	134	(17.1)	**0.001**	67	(12.2)	65	(11.8)	0.853
Stroke	183	(13.2)	145	(18.5)	**0.001**	86	(15.6)	107	(19.4)	0.096
Liver cirrhosis	23	(1.7)	24	(3.1)	**0.031**	9	(1.6)	16	(2.9)	0.157
Malignancy	215	(15.5)	118	(15.1)	0.769	79	(14.3)	79	(14.3)	1.000
COPD	155	(11.2)	103	(13.1)	0.179	64	(11.6)	72	(13.1)	0.464
**Cardiovascular risk factors**, *n* (%)										
Arterial hypertension	977	(70.5)	711	(90.7)	**0.001**	497	(90.2)	490	(88.9)	0.490
Hyperlipidemia	356	(25.7)	300	(38.3)	**0.001**	202	(36.7)	196	(35.6)	0.707
Smoking										
Current	285	(20.6)	117	(14.9)	**0.001**	105	(19.1)	93	(16.9)	0.346
Former	228	(16.5)	157	(20.0)	**0.037**	105	(19.1)	101	(18.3)	0.757
Family history	137	(9.9)	64	(8.2)	0.182	47	(8.5)	48	(8.7)	0.915
**Comorbidities at index hospitalization**, *n* (%)										
Acute coronary syndrome										
Unstable angina	63	(4.5)	36	(4.6)	0.963	32	(5.8)	30	(5.4)	0.794
STEMI	129	(9.3)	46	(5.9)	**0.005**	45	(8.2)	41	(7.4)	0.653
NSTEMI	153	(11.0)	120	(15.3)	**0.004**	70	(12.7)	71	(12.9)	0.928
Acute decompensated heart failure	253	(18.3)	226	(28.8)	**0.001**	137	(24.9)	139	(25.2)	0.889
Atrial fibrillation	571	(41.2)	343	(43.8)	0.253	228	(41.4)	236	(42.8)	0.625
Stroke	187	(13.5)	111	(14.2)	0.670	76	(13.8)	79	(14.3)	0.795
**Medication at index admission**, *n* (%)										
ACE inhibitor	434	(31.3)	336	(42.9)	**0.001**	209	(37.9)	220	(39.9)	0.497
ARB	273	(19.7)	210	(26.8)	**0.001**	138	(25.0)	157	(28.5)	0.196
Beta blocker	713	(51.5)	512	(65.3)	**0.001**	347	(63.0)	351	(63.7)	0.803
Aldosterone antagonist	118	(8.5)	86	(11.0)	0.060	60	(10.9)	56	(10.2)	0.695
ARNI	13	(0.9)	6	(0.8)	0.677	6	(1.1)	3	(0.5)	0.315
SGLT2 inhibitor	2	(0.1)	43	(5.5)	**0.001**	1	(0.2)	29	(5.3)	**0.001**
Loop diuretics	412	(29.7)	399	(50.9)	**0.001**	207	(37.6)	254	(46.1)	**0.004**
Statin	529	(38.2)	450	(57.4)	**0.001**	285	(51.7)	303	(55.0)	0.277

ACE, angiotensin-converting enzyme; ARB, angiotensin receptor blocker; ARNI, angiotensin receptor neprilysin inhibitor; COPD, chronic obstructive pulmonary disease; ICD, implantable cardioverter defibrillator; CRT-D, cardiac resynchronization therapy with defibrillator; DBP, diastolic blood pressure; IQR, interquartile range; (N)STEMI, non-ST-segment elevation myocardial infarction; SBP, systolic blood pressure; SGLT2, sodium glucose linked transporter 2; s-ICD, subcutaneous implantable cardioverter defibrillator. Level of significance: *p* ≤ 0.05. Bold type indicates statistical significance.

**Table 2:** Heart failure-related and procedural data.

	Without Propensity Score Matching					With Propensity Score Matching				
	Non-Diabetics (*n* = 1385)		Diabetics (*n* = 784)		*p*-Value	Non-Diabetics (*n* = 551)		Diabetics (*n* = 551)		*p*-Value
**Heart failure etiology**, *n* (%)										
Ischemic cardiomyopathy	741	(53.5)	509	(64.9)	**0.001**	348	(63.2)	343	(62.3)	0.473
Non-ischemic cardiomyopathy	104	(7.5)	42	(5.4)		29	(5.3)	34	(6.2)	
Hypertensive cardiomyopathy	119	(8.6)	57	(7.3)		56	(10.2)	42	(7.6)	
Congenital heart disease	3	(0.2)	1	(0.1)		1	(0.2)	0	(0.0)	
Valvular heart disease	66	(4.8)	30	(3.8)		19	(3.4)	23	(4.2)	
Tachycardia-associated	30	(2.2)	8	(1.0)		18	(3.3)	13	(2.4)	
Tachymyopathy	67	(4.8)	23	(2.9)		10	(1.8)	7	(1.3)	
Pacemaker-induced cardiomyopathy	13	(0.9)	6	(0.8)		6	(1.1)	6	(1.1)	
Unknown	242	(17.5)	108	(13.8)		64	(11.6)	83	(15.1)	
**NYHA functional class**, *n* (%)										
I/II	1063	(76.7)	514	(65.5)	**0.001**	398	(72.2)	380	((69.0)	0.246
III	218	(15.7)	187	(23.9)		95	(17.2)	121	(22.0)	
IV	104	(7.5)	83	(10.6)		58	(10.5)	50	(9.1)	
**Echocardiographic data**										
LVEF, %, median (IQR)	45 (45–47)		45 (45–47)		0.128	45 (45–47)		45 (45–47)		0.863
IVSd, median (IQR)	12 (10–13)		12 (11–13)		**0.001**	12 (11–13)		12 (11–13)		**0.024**
TAPSE, mm, median (IQR)	20 (17–23)		20 (17–23)		0.152	20 (18–23)		20 (17–23)		0.325
Diastolic dysfunction, *n* (%)	971	(70.1)	590	(75.3)	**0.010**	419	(76.0)	408	(74.0)	0.444
Moderate–severe aortic stenosis, *n* (%)	127	(9.2)	86	(11.0)	0.176	59	(10.7)	59	(10.7)	1.000
Moderate–severe aortic regurgitation, *n* (%)	56	(4.0)	27	(3.4)	0.484	27	(4.9)	21	(3.8)	0.376
Moderate–severe mitral regurgitation, *n* (%)	174	(12.6)	86	(11.0)	0.272	68	(12.3)	67	(12.2)	0.927
Moderate–severe tricuspid regurgitation, *n* (%)	233	(16.8)	110	(14.0)	0.087	87	(15.8)	87	(15.8)	1.000
**Coronary angiography**, *n* (%)	570	(41.2)	323	(41.2)	0.984	247	(44.8)	234	(42.5)	0.430
No evidence of coronary artery disease	129	(22.6)	42	(13.0)	**0.001**	43	(17.4)	34	(14.5)	**0.008**
1-vessel disease	119	(20.9)	47	(14.6)		56	(22.7)	39	(16.7)	
2-vessel disease	137	(24.0)	54	(16.7)		55	(22.3)	37	(15.8)	
3-vessel disease	185	(32.5)	180	(55.7)		93	(37.7)	124	(53.0)	
CABG	31	(5.4)	40	(12.4)	**0.001**	25	(10.1)	26	(11.1)	0.725
Chronic total occlusion	54	(9.5)	59	(18.3)	**0.001**	26	(10.5)	36	(15.4)	0.112
PCI, *n* (%)	311	(54.6)	169	(52.3)	0.519	141	(57.1)	120	(51.3)	0.202
Sent to CABG, *n* (%)	20	(3.5)	31	(9.6)	**0.001**	9	(3.6)	23	(9.8)	**0.007**
**Baseline laboratory values**, median (IQR)										
Creatinine, mg/dL	1.02 (0.83–1.32)		1.20 (0.95–1.67)		**0.001**	1.10 (0.91–1.48)		1.12 (0.91–1.49)		0.604
eGFR, mL/min/1.73 m^2^	70 (50–90)		58 (39–77)		**0.001**	64 (43–83)		62 (44–80)		0.617
Hemoglobin, g/dL	12.6 (10.6–14.1)		12.0 (10.3–13.7)		**0.001**	12.4 (10.4–14.0)		12.3 (10.5–13.8)		0.837
HbA1c, %	5.6 (5.3–5.8)		7.0 (6.4–8.0)		**0.001**	5.7 (5.3–5.9)		7.0 (6.3–7.9)		**0.001**
LDL-cholesterol, mg/dL	102 (78–130)		91 (68–121)		**0.001**	97 (76–126)		91 (70–121)		0.098
HDL-cholesterol, mg/dL	44 (35–54)		39 (32–46)		**0.001**	43 (34–53)		39 (32–46)		**0.001**
C-reactive protein, mg/L	13 (3–43)		14 (4–45)		0.077	11.8 (2.9–41.7)		12.5 (3.8–42.2)		0.367
NT-pro BNP, pg/mL	2283 (774–5454)		3487 (1551–7958)		**0.001**	2375 (963–7313)		3001 (1417–7431)		0.176
Cardiac troponin I, µg/L	0.03 (0.02–0.16)		0.03 (0.02–0.21)		0.130	0.03 (0.02–0.17)		0.03 (0.02–0.17)		0.885
**Medication at discharge**, *n* (%)										
ACE inhibitor	676	(50.3)	375	(49.9)	0.837	271	(50.5)	256	(48.4)	0.499
ARB	277	(20.6)	216	(28.7)	**0.001**	135	(25.1)	166	(31.4)	**0.024**
Beta blocker	1032	(76.8)	591	(78.6)	0.358	438	(81.6)	410	(77.5)	0.100
Aldosterone antagonist	172	(12.8)	120	(16.0)	**0.046**	90	(16.8)	77	(14.6)	0.322
ARNI	17	(1.3)	8	(1.1)	0.683	8	(1.5)	3	(0.6)	0.136
SGLT2 inhibitor	15	(1.1)	67	(8.9)	**0.001**	10	(1.9)	49	(9.3)	**0.001**
Loop diuretics	541	(40.3)	467	(62.1)	**0.001**	270	(50.3)	300	(56.7)	**0.035**
Statin	868	(64.6)	566	(75.3)	**0.001**	396	(73.7)	395	(74.7)	0.730
Insulin	0	(0.0)	286	(38.0)	**0.001**	0	(0.0)	200	(37.8)	**0.001**
Metformin	0	(0.0)	276	(36.7)	**0.001**	0	(0.0)	202	(38.2)	**0.001**
DPP4 inhibitors	0	(0.0)	200	(26.6)	**0.001**	0	(0.0)	158	(30.0)	**0.001**
GLP1 analogues	0	(0.0)	13	(1.7)	**0.001**	0	(0.0)	8	(1.5)	**0.001**

ACE, angiotensin-converting enzyme; ARB, angiotensin receptor blocker; ARNI, angiotensin receptor neprilysin inhibitor; CABG, coronary artery bypass grafting; DPP4, dipeptidyl-peptidase 4; eGFR, estimated glomerular filtration rate; GLP1, glucagon-like peptide; HbA1c, glycated hemoglobin; HDL, high-density lipoprotein; IQR, interquartile range; IVSd, interventricular septal end diastole; LDL, low-density lipoprotein; LVEF, left ventricular ejection fraction; NT-pro BNP, aminoterminal pro-B-type natriuretic peptide; NYHA, New York Heart Association; PCI, percutaneous coronary intervention; SGLT2, sodium glucose linked transporter 2; TAPSE, tricuspid annular plane systolic excursion. Level of significance: *p* ≤ 0.05. Bold type indicates statistical significance.

**Table 3:** Follow-up data, primary and secondary endpoints.

	**Without Propensity Score Matching**						
	**Non-Diabetics ** **(*n* = 1385)**		**Diabetics ** **(*n* = 784)**		**HR**	**95% CI**	***p*-Value**
**Primary endpoint**, *n* (%)							
All-cause mortality, at 30 months	396	(28.6)	281	(35.8)	1.273	1.092–1.483	**0.002**
**Secondary endpoints**, *n* (%)							
All-cause mortality, in-hospital	42	(3.0)	32	(4.1)	1.094	0.688–1.740	0.704
Heart failure-related rehospitalization, at 30 months	144	(10.7)	134	(17.8)	1.714	1.355–2.169	**0.001**
Cardiac rehospitalization, at 30 months	266	(19.8)	193	(25.7)	1.334	1.108–1.606	**0.002**
Revascularization, at 30 months	77	(5.7)	63	(8.4)	1.467	1.058–2.059	**0.022**
Acute myocardial infarction, at 30 months	29	(2.2)	35	(4.7)	2.169	1.326–3.548	**0.002**
Stroke, at 30 months	38	(2.8)	19	(2.5)	0.881	0.508–1.528	0.652
MACCE, at 30 months	488	(35.2)	346	(44.1)	1.298	1.131–1.489	**0.001**
**Follow-up data,** median (IQR)							
Hospitalization time, days	8 (5–14)		10 (6–17)		-	-	**0.001**
ICU time, days	0 (0–1)		0 (0–1)		-	-	0.216
Follow-up time, days	917 (389–1691)		888 (334–1561)		-	-	0.164
	**With propensity score matching**						
	**Non-Diabetics ** **(*n* = 551)**		**Diabetics ** **(*n* = 551)**		**HR**	**95% CI**	***p*-Value**
**Primary endpoint**, *n* (%)							
All-cause mortality, at 30 months	147	(26.7)	182	(33.0)	1.265	1.018–1.572	**0.034**
**Secondary endpoints**, *n* (%)							
All-cause mortality, in-hospital	14	(2.5)	22	(4.0)	1.077	0.874–1.245	0.813
Heart failure-related rehospitalization, at 30 months	78	(14.5)	89	(16.8)	1.172	0.865–1.589	0.306
Cardiac rehospitalization, at 30 months	131	(24.4)	128	(24.2)	1.018	0.987–1.188	0.904
Revascularization, at 30 months	41	(7.6)	40	(7.6)	0.982	0.635–1.518	0.934
Acute myocardial infarction, at 30 months	17	(3.2)	19	(3.6)	1.132	0.588–2.178	0.710
Stroke, at 30 months	16	(3.0)	14	(2.6)	0.876	0.428–1.796	0.718
MACCE, at 30 months	194	(35.2)	229	(41.6)	1.210	1.000–1.465	**0.050**
**Follow-up data**, median (IQR)							
Hospitalization time, days	8 (5–15)		9 (6–16)		-	-	0.205
ICU time, days	0 (0–1)		0 (0–1)		-	-	0.789
Follow-up time, days	880 (432–1661)		915 (346–1654)		-	-	0.778

CI, confidence interval; HR, hazard ratio; ICU, intensive care unit; MACCEs, major adverse cardiac and cerebrovascular events. Level of significance: *p* ≤ 0.05. Bold type indicates statistical significance.

**Table 4:** Multivariable Cox regression analyses in diabetics and non-diabetics with regard to all-cause mortality and heart failure-related rehospitalization at 30 months.

	All-Cause Mortality			Heart Failure-Related Rehospitalization		
	HR	95% CI	*p*-Value	HR	95% CI	*p*-Value
**Diabetics**						
Age	1.041	1.022–1.060	**0.001**	1.003	0.981–1.025	0.798
Male	1.150	0.841–1.574	0.382	0.756	0.505–1.134	0.176
Body mass index	0.970	0.938–1.002	0.065	0.992	0.953–1.032	0.681
Heart rate	1.002	0.994–1.010	0.686	1.003	0.993–1.013	0.618
Prior congenital heart failure	1.122	0.787–1.599	0.526	1.663	1.016–2.721	**0.043**
Prior decompensation	0.862	0.544–1.367	0.528	1.710	1.003–2.915	**0.049**
Creatinine	1.204	1.081–1.342	**0.001**	1.171	1.020–1.344	**0.025**
Hemoglobin	0.887	0.822–0.957	**0.002**	0.973	0.883–1.071	0.572
Arterial hypertension	0.735	0.409–1.319	0.302	1.664	0.600–4.613	0.328
Hyperlipidemia	0.908	0.667–1.236	0.539	0.767	0.510–1.153	0.202
Acute myocardial infarction	0.959	0.647–1.422	0.836	1.350	0.796–2.288	0.265
Acute decompensated heart failure	1.448	1.048–2.001	**0.025**	1.880	1.232–2.867	**0.003**
Atrial fibrillation	1.273	0.914–1.772	0.153	2.188	1.386–3.455	**0.001**
TAPSE < 18 mm	0.967	0.932–1.003	0.073	0.984	0.948–1.021	0.394
**Non-Diabetics**						
Age	1.041	1.028–1.054	**0.001**	1.013	0.995–1.031	0.161
Male	1.394	1.073–1.810	**0.013**	1.217	0.810–1.829	0.344
Body mass index	0.939	0.911–0.968	**0.001**	1.045	1.013–1.078	**0.005**
Heart rate	0.996	0.990–1.002	0.201	1.003	0.995–1.011	0.480
Prior congenital heart failure	1.337	0.995–1.796	0.054	1.942	1.240–3.042	**0.004**
Prior decompensation	0.836	0.549–1.273	0.404	1.366	0.806–2.312	0.246
Creatinine	0.984	0.892–1.085	0.743	0.992	0.837–1.176	0.929
Hemoglobin	0.777	0.733–0.825	**0.001**	0.860	0.786–0.942	**0.001**
Arterial hypertension	0.999	0.740–1.349	0.995	1.198	0.720–1.994	0.486
Hyperlipidemia	0.442	0.315–0.618	**0.001**	1.048	0.685–1.604	0.828
Acute myocardial infarction	0.637	0.435–0.930	**0.020**	0.622	0.314–1.231	0.173
Acute decompensated heart failure	1.748	1.335–2.290	**0.001**	1.892	1.254–2.856	**0.002**
Atrial fibrillation	1.190	0.903–1.567	0.217	1.463	0.947–2.261	0.087
TAPSE < 18 mm	1.003	0.993–1.014	0.519	0.974	0.932–1.017	0.233

CI, confidence interval; HR, hazard ratio; TAPSE, tricuspid annular plane systolic excursion. Level of significance: *p* ≤ 0.05. Bold type indicates statistical significance.

**Table 5:** Multivariable Cox regression analyses in diabetics with regard to all-cause mortality and heart failure-related rehospitalization at 30 months.

	All-Cause Mortality			Heart Failure-Related Rehospitalization		
	HR	95% CI	*p*-Value	HR	95% CI	*p*-Value
Age	1.054	1.038–1.070	**0.001**	1.010	0.991–1.028	0.300
Male	1.190	0.909–1.559	0.205	0.808	0.565–1.156	0.244
Prior coronary artery disease	0.777	0.514–1.175	0.232	1.525	0.880–2.644	0.132
Prior myocardial infarction	1.142	0.790–1.649	0.481	1.109	0.705–1.732	0.655
Arterial hypertension	0.729	0.464–1.145	0.170	1.444	0.663–3.148	0.355
Hyperlipidemia	1.074	0.822–1.404	0.601	0.683	0.470–0.993	**0.046**
eGFR	0.996	0.990–1.002	0.178	0.988	0.980–0.996	**0.005**
Ischemic cardiomyopathy	0.898	0.624–1.292	0.562	1.069	0.628–1.822	0.805
NYHA functional class	1.129	1.000–1.275	0.049	1.546	1.306–1.830	**0.001**
SGLT2 inhibitor	0.552	0.270–1.130	0.104	0.661	0.286–1.527	0.333
Insulin	1.332	1.018–1.742	**0.037**	1.208	0.841–1.734	0.307
Metformin	0.805	0.587–1.102	0.176	1.719	1.119–2.639	**0.013**
DPP4 inhibitor	1.158	0.877–1.528	0.301	1.026	0.689–1.508	0.896

CI, confidence interval; HR, hazard ratio; NYHA, New York Heart Association; SGLT2, sodium glucose linked transporter 2; DPP4, dipeptidyl-peptidase 4. Level of significance: *p* ≤ 0.05. Bold type indicates statistical significance.

## Data Availability

The datasets used and/or analyzed during the current study are available from the corresponding author upon reasonable request.
